# The Mammalian Olfactory Bulb Contributes to the Adaptation of Odor Responses: A Second Perceptual Computation Carried Out by the Bulb

**DOI:** 10.1523/ENEURO.0322-21.2021

**Published:** 2021-09-23

**Authors:** Douglas A. Storace, Lawrence B. Cohen

**Affiliations:** 1 Department of Cellular and Molecular Physiology, Yale University School of Medicine, New Haven, CT 06520; 2 Department of Biological Science, Florida State University, Tallahassee, FL 32306; 3 Brain Science Institute, Korea Institute of Science and Technology, Seoul 136-791, Republic of Korea

**Keywords:** two-photon, calcium imaging, epifluorescence, mitral/tufted cells, olfactory bulb, olfactory receptor neuron

## Abstract

While humans and other mammals exhibit adaptation to odorants, the neural mechanisms and brain locations involved in this process are incompletely understood. One possibility is that it primarily occurs as a result of the interactions between odorants and odorant receptors on the olfactory sensory neurons in the olfactory epithelium. In this scenario, adaptation would arise as a peripheral phenomenon transmitted to the brain. An alternative possibility is that adaptation occurs because of processing in the brain. We made an initial test of these possibilities using a two-color imaging strategy to simultaneously measure the activity of the olfactory receptor nerve terminals (input to the bulb) and mitral/tufted cell apical dendrites (output from the bulb) in anesthetized and awake mice. Repeated odor stimulation at the same concentration resulted in a decline in the bulb output, while the input remained relatively stable. Thus, the mammalian olfactory bulb appears to participate in generating the perception of olfactory adaptation under this stimulus condition. Similar experiments conducted previously showed that the bulb may also participate in the perception of concentration invariance of odorant recognition (Storace and Cohen, 2017); thus, the bulb is simultaneously carrying out more than one computation, as is true of other mammalian brain regions and perhaps is the case for all animals with sophisticated nervous systems. However, in contrast with other sensory systems (Van Essen et al., 1992), the very first processing stage in the olfactory system has an output that may directly represent perceptions.

## Significance Statement

The ability to identify sensory information embedded in a complex environment requires the brain to be able to filter background information and attend to novel stimuli. Adaptation is likely to be important in this process as it allows neurons in a brain area to adjust to the broader sensory environment. However, the brain area(s) that are involved in olfactory adaptation remain an outstanding question. We conducted simultaneous imaging of the input to the olfactory bulb, and the output from the bulb to the rest of the brain. This comparison revealed that the sensory input from the nose provides a relatively stable representation of the odor environment, whereas robust adaptation occurred in the bulb output. This is the second perceptual calculation, carried out simultaneously, by the olfactory bulb.

## Introduction

The ability to identify sensory information embedded in a complex environment requires the brain to be able to filter background information and attend to novel stimuli (Linster et al., 2007; Gottfried, 2010). This is a challenging problem in the olfactory system because odor perception is thought to be related to the combination of activated olfactory receptors, yet different receptors can be activated by the same odor at different concentrations and the same receptor can be activated by perceptually different odors (Duchamp-Viret et al., 1999; Wachowiak and Cohen, 2001; Hu et al., 2020). A new and potentially important odor stimulus can activate some of the same odor receptors as those activated by a background odor. Thus, distinguishing a novel odor amid a background is inherently confounded at the receptor level. In principle, this confound could be obviated by adaptation, a process in which the input-output transformation of a neuron or brain area is not static, but changes based on repeated presentation of the same odor. Adaptation is essential for filtering out background information (Barlow, 1961; Wark et al., 2007; Weber and Fairhall, 2019). It is well established that animals exhibit habituation to odor stimuli, a phenomena in which there are reduced behavioral responses to repetitive or constant odor stimulation (Rankin et al., 2009; Pellegrino et al., 2017). However, the brain areas and neural mechanisms involved in this perception are incompletely understood.

Olfactory receptor neurons, which send their projections to the olfactory bulb, adapt in response to some (but not all) stimulus conditions (Kurahashi and Menini, 1997; Leinders-Zufall et al., 1998; Zufall and Leinders-Zufall, 2000; Reisert and Matthews, 2001a; Verhagen et al., 2007; Lecoq et al., 2009; Ghatpande and Reisert, 2011). Similar observations have been made in the mitral and tufted cells, which receive olfactory receptor neuron input, and project out of the bulb. There are reports of strongly adapting mitral/tufted cells (Margrie et al., 2001; Dietz and Murthy, 2005; McNamara et al., 2008; Chaudhury et al., 2010; Ogg et al., 2015), while others indicate that they do not adapt and instead strongly adapting neurons are found in their cortical targets (Sobel and Tank, 1993; Wilson, 2000a,b; Cang and Isaacson, 2003; Kadohisa and Wilson, 2006; Shea et al., 2008).

These different experimental outcomes likely reflect differences in preparation type and/or stimulus paradigms as well as the fact that adaptation is mediated by different processes at different timescales (McNamara et al., 2008; Chaudhury et al., 2010; Cafaro, 2016). Indeed adaptation can occur presynaptically at the olfactory receptor neuron synapse (Murphy et al., 2004; Kazama and Wilson, 2008). This would presumably result in inherited adaptation in their postsynaptic targets. Adaptation could also occur via circuit mechanisms (Margrie et al., 2001; Dietz and Murthy, 2005), in which case mitral/tufted cell adaptation could occur in the absence of changes in olfactory receptor neuron input. Thus, although adaptation has been described in mitral/tufted cells, it is impossible to know whether the olfactory bulb was involved or whether the changes are inherited without knowing the olfactory receptor neuron input.

Here, we sought to determine whether it is possible to disentangle these issues by simultaneously measuring the input-output transformation of the bulb (Wilson et al., 2004; Storace and Cohen, 2017). A simultaneous measurement of the input and output makes it certain that the animal condition, odorant delivery, anesthetic level, respiration rate, and other external and internal variables are the same for both measurements. The glomerular input and output odor activation patterns were measured and compared in response to repeated three second odorant presentations separated by six second intervals. In many experiments input and output were measured simultaneously in awake or anesthetized mice using wide-field epifluorescence imaging. In other experiments, input and output were measured in separate, anesthetized preparations using two-photon imaging. The results demonstrate a specific odor presentation paradigm in which the olfactory receptor neurons respond with a relatively stable representation of the sensory environment, while olfactory bulb processing results in output adaptation. Thus, the olfactory bulb may also contribute to sensory adaptation.

## Materials and Methods

### Surgery and imaging in adult mice

All experiments were performed in accordance with relevant guidelines and regulations, including a protocol approved by the Institutional Animal Care and Use Committee at Yale University. Thy1-GCaMP6f GP5.11 transgenic mice were acquired from Jax (stock #024339; Dana et al., 2014). Tbx21-Cre (Jax #024507), and OMP-Cre (Jax #006668; Li et al., 2004) transgenic mice were acquired from Jax and crossed to a floxed GCaMP6f reporter transgenic mouse (Jax #024105; Madisen et al., 2015). OMP-GCaMP3 (Jax #029581) transgenic mice were a gift from Tom Bozza, which were crossed to the Tbx21-Cre line to create a new line in which GCaMP3 was expressed in the olfactory receptor neurons, and cre recombinase was expressed in the mitral/tufted cells (OMP-GCaMP3 x Tbx21-Cre). In this line, the output cells could be targeted with a cre-dependent adeno-associated virus (AAV). Experiments with OMP-GCaMP3 mice were made in animals that were heterozygous. Animals used in the study were confirmed to express the appropriate transgenes via genotyping by Transnetyx. Mice were housed under standard environmental conditions under a 12h light/dark cycle. Our measurements were made during the light phase.

For all surgical procedures, male or female adult (40–100 d old) mice were anesthetized with a mixture of ketamine (90 mg kg^−1^) and xylazine (10 mg kg^−1^). Anesthesia was supplemented as needed to maintain areflexia, and anesthetic depth was monitored periodically via the pedal reflex. Animal body temperature was maintained at ∼37.5°C using a heating pad placed underneath the animal. For recovery manipulations, animals were maintained on the heating pad until awakening. Local anesthetic (1% bupivacaine, McKesson Medical) was applied to all incisions. Respiration was recorded with a piezoelectric sensor.

Calcium dye (Cal-590 Dextran, #20509, AAT Bioquest) was loaded into olfactory sensory neurons of mice via the time-honored, yet thorny approach described by Wachowiak and Cohen (2001). Mice were anesthetized, placed on their back, and an 8-μl mixture of 4%/0.2% calcium dye/Triton X-100 was drawn into a Hamilton syringe with a flexible plastic tip, which was inserted ∼10 mm into the nasal cavity; 2 μl of the dye/Triton X-100 mixture was infused into the nose every 3 min. Mice were allowed to recover for at least 4 d before optical measurements. The olfactory sensory neurons were also measured using the genetically encoded calcium indicators (GECIs) GCaMP3 and GCaMP6f (Tian et al., 2009; Chen et al., 2013). The mitral/tufted cells were measured using GCaMP6f, jRGECO1a, and jRCaMP1a (Chen et al., 2013; Dana et al., 2014, 2016). GCaMP6f was endogenously expressed in mitral/tufted cells in Thy1-GCaMP6f 5.11 and Tbx21-Cre x Flex-GCaMP6f transgenic mice. Cre-dependent AAVs were used to express jRGECO1a and in Tbx21-Cre (or OMP-GCaMP3 x Tbx21-Cre) transgenic mice.

For epifluorescence imaging, a custom headpost was attached to the top of the posterior skull using metabond (Parkell). The skull above the dorsal olfactory bulb was thinned and covered with an optically transparent glue and allowed to dry. For recordings in awake mice, the skull was covered with Kwik-Sil (WPI), and the mouse was allowed to recover for 3 d. The recording chamber was a conical vial with the sealed end cut open in which the mouse was allowed to acclimate for a minimum of 3 d before imaging experiments. Mice were initially placed in the vial for periods of up to 10 min, which gradually increased up to 30 min. Animals did not exhibit prolonged struggling or other signs of distress. Anesthetized recordings were conducted on the same day as the dissection while being maintained on the heating pad at 37.5°C, and with anesthetic depth being monitored frequently via pedal reflex.

Epifluorescence imaging was done using a microscope made from Thorlabs components with two Prizmatix LEDs (UHP-T-LED-455 and UHP-T-LED-545), which were filtered with a 488/10-nm (Semrock FF01-488/10), and a 575/5-nm (Semrock FF01-575/5) excitation filter, respectively. In some preparations the 488/10 filter was substituted with a 470/40-nm, or 461/5-nm filter. A beam combiner dichroic cube directed the light from both LEDs toward a dual-reflectance, dual emission dichroic mirror (59009bs; Chroma) that reflected the excitation light toward the preparation, while transmitting the fluorescence emission of both fluorophores in the emission pathway. The fluorescence emission passed through a 175-mm focal length lens (Edmund Optic), and a dual bandpass filter (Chroma 59009m) before being recorded with a NeuroCCD-SM256 camera with 2 × 2 (128 × 128 pixels) or 3 × 3 (84 × 84 pixels) binning at frame rates between 25 and 50 Hz using NeuroPlex software (RedShirtImaging).

A schematic of the LED strobing is included in Extended Data Figure 1-1, which was conducted by sending a trigger that defined the onset of each camera frame to an Arduino Mega 2560, which was programmed to send alternating 5 V triggers to the two LED power supplies. The precision of the Arduino board was measured using an oscilloscope (WaveAce 102, Teledyne LeCroy). The delay from detection of a triggered pulse on the camera pin to the output of either of the LED pins was between 4 and 25 μs. The jitter of the output of the LED pin was similarly small. The board was programmed to include a short buffer (500 μs) at the beginning and end of each camera frame during which the LED was off, to avoid cross talk across frames.

The strobing control analysis in Extended Data Figure 1-1 was performed in a transgenic mouse that had GCaMP6f expressed in the olfactory receptor neurons (OMP-Cre x Ai95.flex.GCaMP6f). In this experiment, odor-evoked signals were measured in one trial while the light was on continuously. In another trial, the blue light was strobed so that it was only on during alternate camera frames. The traces in Extended Data Figure 1-1*C* are from single trials. Similar results were observed in two additional preparations.

The procedures for two-photon imaging experiments were similar to those for epifluorescence. The skull was removed and replaced with agarose and a cover glass window. All imaging took place while the animal was anesthetized on the same day as the surgery. Two-photon imaging was performed with a modified Sutter moveable objective microscope (MOM; Sutter Instruments) equipped with an 8-kHz resonant scanner (Cambridge Technologies). The tissue was illuminated with 940- to 980-nm laser light for GCaMP, or 1040 nm for jRGECO1a using a Coherent Discovery laser (Coherent). Imaging was performed using either a Nikon CFI APO LWD 25 × 1.10 N.A. a Nikon CFI LWD 16 × 0.80 N.A., a Leica 20 × 1.0 NA, an Olympus 10 × 0.6 NA, a Zeiss 10 × 0.5 NA, or an Olympus 20 × 1.0 NA objective. Fluorescence emission passed through a 510/84 bandpass filter and was detected with a GaAsP PMT (Hamamatsu). Power delivered to the sample ranged from 75 to 140 mW as determined using a power meter (Thorlabs PM100D) placed underneath the microscope objective at the beginning of each experiment. Two-photon movies were spatially binned from 512 × 512 to 256 × 256 pixels.

### Data analysis

Odorant-evoked signals were collected in consecutive trials separated by a minimum of 3 min. The individual trials were manually inspected, and occasional trials with obvious motion artifacts were discarded. The mice underwent an initial habituation trial on the day before data collection where the mouse was imaged in response to odor stimulation to confirm that the dye loading or AAV injection was successful and to habituate the mouse to the experimental apparatus. On the data collection day, the total number of recording trials (and hence odor presentations) each mouse experienced varied. The data reflect responses to odor stimuli the animals had already been exposed to.

**Figure 1.**
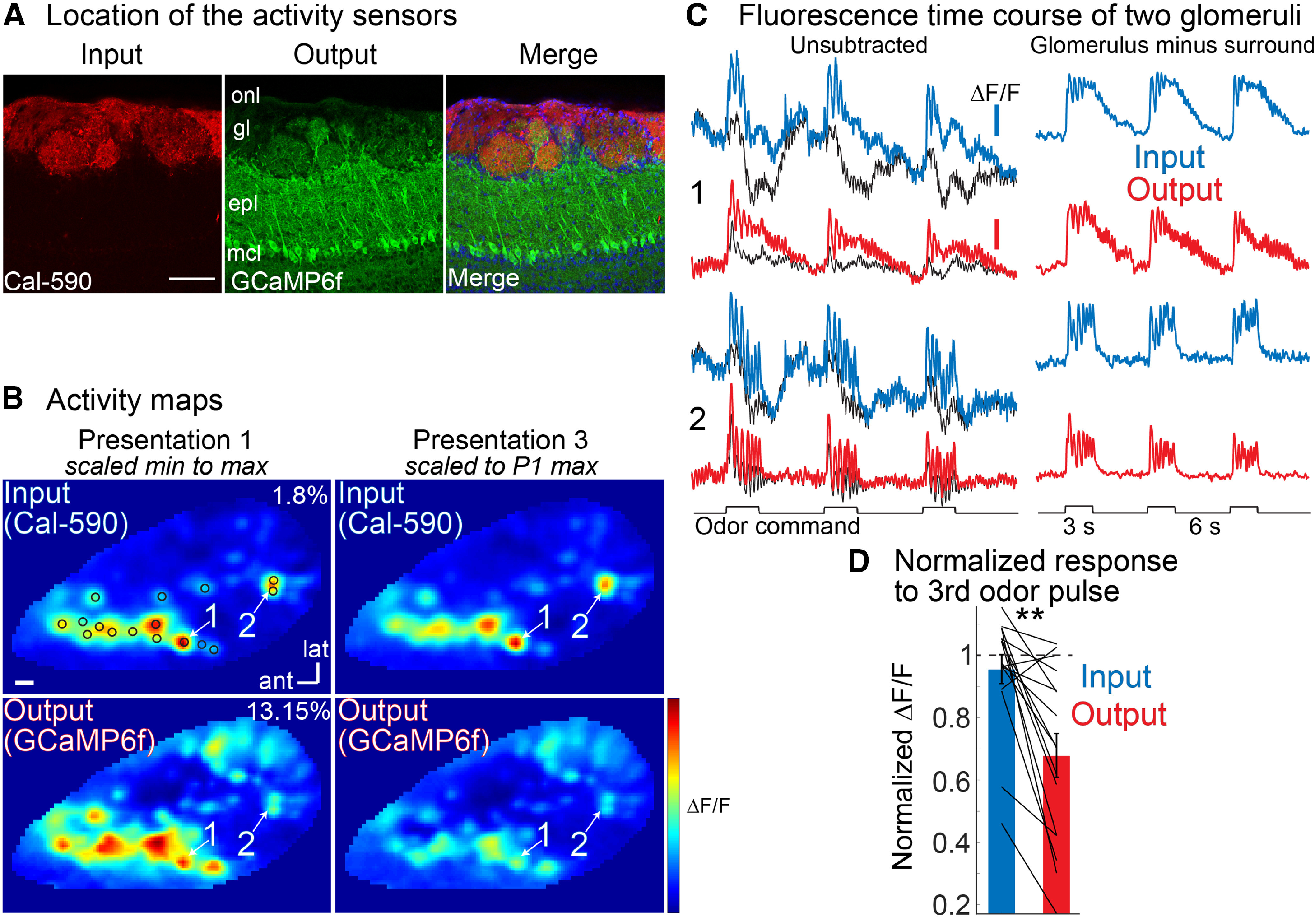
Measuring the olfactory bulb input-output transformation using wide-field imaging reveals a contribution to adaptation. ***A***, Histology illustrating spectrally distinct sensors targeted to input (Cal-590) and output (GCaMP6f). ***B****–****D***, Strobing LEDs were used to image input and output on alternate camera frames in response to repeated odor presentations. Strobing did not noticeably impact the signal-to-noise ratio (Extended Data Fig. 1-1) and the olfactometer delivers repeatable odor pulses (Extended Data Fig. 1-2). ***B***, Input (top, left) and output (bottom, left) activity maps are shown in response to the first odor presentation scaled to their minimum and maximum ΔF/F values (max ΔF/F indicated on panel). The activity map evoked by the third odor pulse (right panels) is shown with the color scale set to the maximum ΔF/F value evoked by the first odor pulse. ***C***, Fluorescence time course of input and output for two glomeruli from one of the single trials used to generate the activity maps in panel ***B***. Unsubtracted, The colored traces are from the center of the activated glomeruli. The signal from the immediate surround is shown as the superimposed thin black trace. Glomerulus minus surround, Fluorescence traces that have had the surround subtracted from the glomerular center. The odor used was methyl valerate at 2% of saturated vapor. ***D***, Quantification of the response to the third odor presentation normalized to the first odor presentation (*N* = 16 glomeruli). The dashed line represents the result for zero adaptation; **p < 0.005. The scale bars in panels ***A***, ***B*** indicate 100 μm, and the scale bars in panel ***C*** indicate 1% and 5% ΔF/F, respectively. Similar results were obtained using different sensor combinations (Extended Data Figs. 1-3, 1-4, 1-5, 1-6 1-7). onl, olfactory nerve layer; gl, glomerular layer; epl, external plexiform layer; mcl, mitral cell layer; ant, anterior; lat, lateral.

Individual glomeruli were visually identified in the average fluorescence intensity, or via a frame subtraction analysis in NeuroPlex that identified activated glomeruli (Wachowiak and Cohen, 2001; Storace and Cohen, 2017). The frame subtraction analysis color scales (Fig. 1*B*) are scaled to the maximum value of the first odor presentation for input and output, respectively. The maps in panel Figure 1*B* are spatially filtered with one iteration of a 3 × 3 low-pass filter. Response amplitudes were calculated as the difference between the 2-s average around the peak of the response, and the 2 s preceding the stimulus. Fluorescence signals were converted to ΔF/F by dividing the spatial average of the pixels containing each identified glomerulus by the resting fluorescence, and were low-pass filtered at 4 Hz. The resting fluorescence was defined as the average of five frames at the beginning of the trial (epifluorescence imaging) or the average of the 3 s before the first odor presentation (two-photon imaging).

10.1523/ENEURO.0322-21.2021.f1-1 Extended Data Figure 1-1 ***A***, Imaging paradigm for simultaneous measurements from the two fluorophores. ***B***, ***C***, Multiplexing LEDs does not notably impact the signal-to-noise ratio. In one experiment, GCaMP6f was imaged continuously (***B***, top), or was multiplexed so that alternate frames were blank (***C***, bottom). ***C***, Odor-evoked fluorescence signals measured from the illuminated frames were not distinguishable from the continuous recording, and no signal was detectable in the LED-OFF camera frames (black trace). Similar results were obtained in two other preparations. Download Figure 1-1, TIF file.

**Figure 2.**
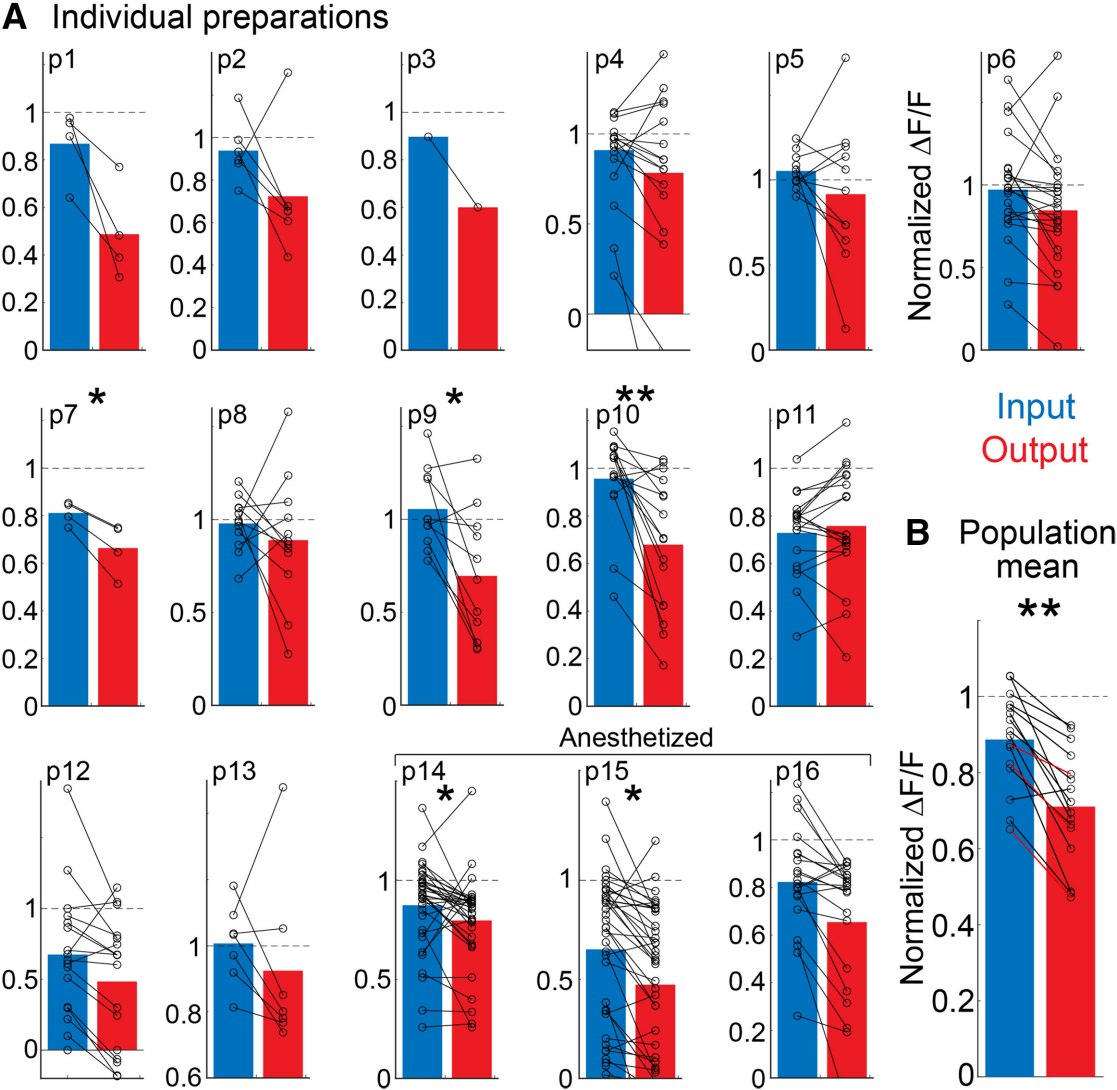
Population summary and individual preparations in which both input and output were measured in the same hemibulbs of awake or anesthetized mice. Measurements are shown as the response to the third odor pulse normalized to the ΔF/F amplitude evoked by the first. ***A***, Signal size measurements from identified glomeruli that were present in both input and output for 16 preparations. Lines show the direction of the same glomeruli for input and output. Preparations 1–13 and 14–16 were conducted in awake and anesthetized mice, respectively. Between 1 and 39 glomeruli were measured in each preparation (15 ± 2.6). Input was measured using Cal-590 dextran or GCaMP3 in 11 and 5 preparations, respectively. Output was measured using GCaMP6f, jRGECO1a, or jRCaMP1a in 11, 3, and 2 preparations, respectively. The sensors and odorant for each preparation are given in Table 1. ***B***, Quantification of signal size across the population of all 16 preparations; *p < 0.05, **p < 0.005.

10.1523/ENEURO.0322-21.2021.f1-2 Extended Data Figure 1-2 The olfactometer delivers repeatable odor pulses. A photoionization detector was used to measure the output of the olfactometer across three odor repeats at two different concentrations. Similar recordings were taken across eight experiments on different days. Download Figure 1-2, TIF file.

**Figure 3.**
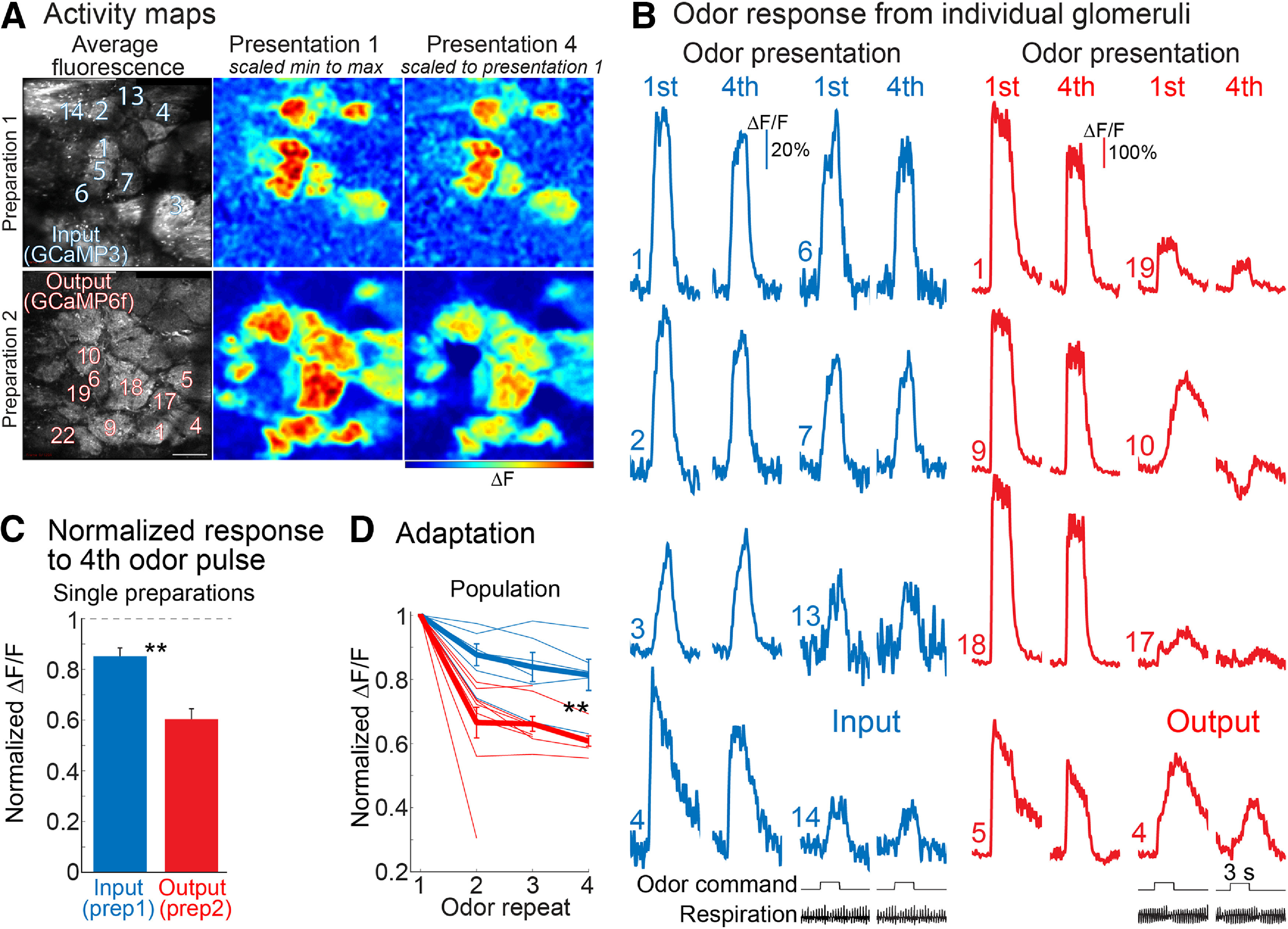
Adaptation of input and output responses measured in different, anesthetized preparations using two-photon imaging. ***A***, Average fluorescence intensity and activity maps from two different preparations in which input (GCaMP3) or output (GCaMP6f) were measured separately. The input maps are relatively similar for the first and fourth odor presentation while the signals in the fourth output map are smaller. Scale bar: 100 μm. ***B***, Fluorescence time courses from activated glomeruli measured in the two preparations in ***A***. The response to the first and fourth odor presentation are cropped and displayed next to each other. The output traces in panel ***B*** are from a single trial, the input traces are from an average of four trials. The heterogeneity of the output responses is further illustrated in Extended Data Figures 3-1, 3-2. ***C***, Quantification of the signal size of the fourth odor presentation normalized to the amplitude evoked by the first odor presentation for all of the glomeruli identified in these two recording trials. The dashed line represents the result for zero adaptation. ***D***, Population summary of adaptation of input and output. Each thin line is from a different preparation. The input and output recordings in ***A***, ***B*** were measured in response to ethyl tiglate (2% saturated vapor) and methyl valerate (2% saturated vapor), respectively; **p < 0.005.

10.1523/ENEURO.0322-21.2021.f1-3 Extended Data Figure 1-3 Histology from three different transgenic mice showing expression of the sensors in the expected locations. ***A***, Tbx21-Cre x Ai95.Flex.GCaMP6f transgenic mouse resulted in mitral/tufted cell-specific expression (middle panel). ***B***, OMP-GCaMP3 x Tbx21-Cre transgenic mouse. GCaMP3 is targeted to the bulb input (left panel). jRGECO1a was expressed in the mitral/tufted cells using a cre-dependent AAV (middle panel). Download Figure 1-3, TIF file.

10.1523/ENEURO.0322-21.2021.f1-4 Extended Data Figure 1-4 The bulb output adapts more than the input in response to repeated odor stimulation in a Tbx21-Cre x Ai95.Flex.GCaMP6f transgenic mouse that had Cal-590 loaded into its OSNs. The data display arrangement and legend are otherwise identical to Figure 2. The odor used was isoamyl acetate at 2% of saturated vapor. The input and output scale bars in panel ***B*** indicate 0.5% and 2% ΔF/F, respectively. Download Figure 1-4, TIF file.

10.1523/ENEURO.0322-21.2021.f1-5 Extended Data Figure 1-5 The bulb output adapts more than the input in response to repeated odor stimulation in an OMP-GCaMP3 x Tbx21-Cre transgenic mouse that was injected with a cre-dependent AAV-expressing jRCaMP1a. The data display arrangement and legend are otherwise identical to Figure 2. The odor used was methyl valerate at 2% of saturated vapor. The input and output scale bars in panel ***B*** indicate 1.8% and 3% ΔF/F, respectively. Download Figure 1-5, TIF file.

10.1523/ENEURO.0322-21.2021.f1-6 Extended Data Figure 1-6 The bulb output adapts more than the input in response to repeated odor stimulation in an OMP-GCaMP3 x Tbx21-Cre transgenic mouse that was injected with a cre-dependent AAV-expressing jRGECO1a. The data display arrangement and legend are otherwise identical to Figure 2. The odor used was methyl valerate at 2% of saturated vapor. The traces in panel ***B*** are from aligned averages. The input and output scale bars in panel ***B*** indicate 2% and 2% ΔF/F, respectively. Download Figure 1-6, TIF file.

10.1523/ENEURO.0322-21.2021.f1-7 Extended Data Figure 1-7 Blue light evokes a large increase in jRGECO1a fluorescence emission in the absence of odor presentation. The green LED (573 nm) illuminated the preparation every other camera frame. No response is present while alternate frames are blank. The introduction of blue (470 nm) light on the alternate camera frames causes a very large, slow increase in jRGECO1a fluorescence emission that even more slowly returned to baseline after the blue LED was removed. Download Figure 1-7, TIF file.

**Table 1:** Summary of all preparations in **Figure 2**

Prep	Input sensor	Output sensor	State	% Sat Vapor	Input	Output	Glomeruli	Rank- sum	Z value	*p* value
1	Cal-590	GCaMP6f	Awake	ia (2%)	0.86 ± 0.07	0.48 ± 0.1	4	25	1.87	0.06
2	Cal-590	GCaMP6f	Awake	mv (2%)	0.94 ± 0.06	0.72 ± 0.12	6	51	1.84	0.06
3	Cal-590	GCaMP6f	Awake	et (2%)	0.9	0.6	1	2	0	1
4	OMP-GCaMP3	jRGECO1a	Awake	et (2%)	0.91 ± 0.07	0.78 ± 0.14	17	314	0.55	0.58
5	OMP-GCaMP3	jRGECO1a	Awake	et (2%)	1.05 ± 0.03	0.91 ± 0.12	11	139	0.787	0.43
6	OMP-GCaMP3	jRCaMP	Awake	mv (2%), et (2%), 2 hep (2%)	0.97 ± 0.06	0.84 ± 0.07	23	607	1.45	0.14
7	Cal-590	GCaMP6f	Awake	mv (0.4%)	0.81 ± 0.02	0.66 ± 0.05	4	26	2.16	0.03
8	Cal-590	GCaMP6f	Awake	mv (2%)	0.97 ± 0.04	0.89 ± 0.1	12	168	1.01	0.31
9	Cal-590	GCaMP6f	Awake	mv (2%)	1.05 ± 0.06	0.69 ± 0.1	11	163	2.36	0.01
10	Cal-590	GCaMP6f	Awake	mv (2%)	0.95 ± 0.05	0.68 ± 0.07	16	349	3.18	0.001
11	OMP-GCaMP3	jRCaMP	Awake	et (2%), mv (0.4%)	0.73 ± 0.04	0.75 ± 0.05	19	362	−0.23	0.81
12	Cal-590	GCaMP6f	Awake	mv (2%, 0.4%)	0.67 ± 0.11	0.48 ± 0.1	17	322.5	0.84	0.39
13	OMP-GCaMP3	jRGECO1a	Awake	et (2%)	1.00 ± 0.04	0.92 ± 0.1	7	64	1.4	0.15
14	Cal-590	GCaMP6f	Anesthesia	mv (2%, 0.4%, 0.12%), ia (0.4%)	0.87 ± 0.03	0.79 ± 0.04	34	1371	2.4	0.01
15	Cal-590	GCaMP6f	Anesthesia	mv (2%, 0.4%, 0.12%), 2 hep (2%), ia (2%, 0.4%)	0.65 ± 0.06	0.47 ± 0.06	39	1760	2.1	0.02
16	Cal-590	GCaMP6f	Anesthesia	mv (2%, 0.4%, 0.12%)	0.82 ± 0.05	0.65 ± 0.06	21	513	1.5	0.12
		Summary								
Input	Output	Rank sum	Z value	*p* value						
0.88 ± 0.03	0.71 ± 0.04	345	3.03	0.0024						

Input and output values indicate mean ± SEM. Statistics indicate results from a Wilcoxon rank-sum test. mv, methyl valerate; et, ethyl tiglate; 2hep, 2-heptanone; ia, isoamyl acetate.

The scaled subtraction analysis was used to remove common noise and widespread signals (Fig. 1*C*,*D*; Fig. 2; Extended Data Figs. 1-4, 1-5, 1-6, 3-2). The pixels surrounding an activated glomerulus were selected as the “surround.” We made an effort to select nearby pixels that did not overlay other glomeruli appearing in the activity map. The values used in the quantification (Figs. 1*D*, 2) were generated by subtracting the difference between the center and surround values at the peak of the odor response. The example traces shown as “glomerulus minus surround” in Figure 1*C*, and Extended Data Figs. 1-4, 1-5, 1-6, 3-2 were generated using the scaled subtraction function in NeuroPlex. The behavior of the scaled subtraction traces closely reflected that of the activity maps.

The responses to the first and third odor response were then normalized to the response amplitude evoked by the first odor presentation. The two-photon data were not analyzed using the scaled subtraction analysis since two-photon imaging is influenced less by out of focus fluorescence. The average fluorescence intensity images in Figure 3*A* were generated by taking the average of the entire imaging trials. The figures primarily include single trial recordings to highlight the imaging signal-to-noise ratio, although all activity maps (Figs. 1*B*, 3*A*), and quantitative measurements (Figs. 1*D*, 2, 3*C*,*D*) were conducted in averaged trials. The population analysis in Figures 2*B*, 3*D* averaged all normalized measurements for each preparation. For the two-photon analysis, the normalized response to the second, third and fourth odor presentation were grouped across all preparations for input and output respectively (Fig. 3*D*). Two-sample statistical comparisons were performed using a Mann–Whitney *U* test (rank-sum in MATLAB).

10.1523/ENEURO.0322-21.2021.f3-1 Extended Data Figure 3-1 Heterogeneity of glomerular output responses measured using two-photon and wide-field epifluorescence imaging. The glomerular mitral/tufted cell output exhibited diverse response types not seen in the olfactory sensory neuron input. ***A****–****H***, Two-photon imaging: traces are from single trial measurements. The traces in ***A***, ***B***; **C**, ***D***; and ***E***, ***F*** are different glomeruli from the same imaging trial, but in three different preparations. ***H***, Percentage response types across all two-photon output measurements. ***I–K***, Wide-field epifluorescence imaging: glomerulus ***J*** exhibits a slow rise, and early suppression to the later odor pulses (similar to ***B***, ***D***). Glomerulus ***K*** exhibits a suppressed response to the odor (similar to ***F***, ***G***). The traces in ***I****–****K*** data are from the preparation shown in Figure 1. However, qualitatively similar observations were made in multiple preparations. Download Figure 3-1, TIF file.

10.1523/ENEURO.0322-21.2021.f3-2 Extended Data Figure 3-2 The mitral/tufted output exhibited suppressed signals in response to odor stimulation in regions away from activated glomeruli. Two different preparations are shown from a Thy1-GCaMP6f 5.11 (left) transgenic mouse and a Tbx21-GCaMP6f (right) transgenic mouse. ***A***, Activity maps in response to the initial odor presentation. The stimuli were methyl valerate at 0.12% of saturated vapor (left) and isoamyl acetate at 2% of saturated vapor (right). ***B***, The center response of three glomeruli from each activity map (location indicated in ***A***) along with the signal from the diffuse suppressed area. The glomerular center declined with repeated odor presentation, while the suppressed regions did not notably change. The scale bars in panel ***B*** indicate 10% (left) and 5% (right) ΔF/F, respectively. A, anterior; L, lateral. Download Figure 3-2, TIF file.

The bleaching analysis was conducted by measuring the average fluorescence intensity from the spatial average from all the regions of interest used for that preparation at the beginning of the recording trial (i.e., the mean of frames 6–11) and immediately before the third odor pulse (when the odor response to the second stimulus had recovered). The difference between these two values defined the total bleaching within a trial and was used to generate a correction factor for the last odor pulse.

The analysis conducted to compare input and output using the same sensor in different preparations used four mice in which GCaMP6f was expressed in the olfactory receptor neurons (a cross between OMP-Cre and a GCaMP6f reporter mouse). The olfactory receptor neuron GCaMP6f dataset includes a total of 417 odor-glomerulus pairs were measured in response to three different odors (methyl valerate, *N* = 232; isoamyl acetate, *N* = 124; 2-heptanone, *N* = 61) and concentrations (2% of saturated vapor: *N* = 263; 0.36%: *N* = 110; 0.12%: *N* = 37; 0.06% *N* = 7). The mean result from each preparation was compared with the preparations in Figure 2 and Table 1 that expressed GCaMP6f in the mitral/tufted cells. The comparison of adaptation measured using different input sensors compared the average adaptation in the four preparations in which GCaMP6f was expressed in the olfactory receptor neurons to the preparations in Figure 2 and Table 1 in which the input was measured using Cal-590 (*N* = 11 preparations) and GCaMP3 (*N* = 5 preparations). The statistical comparison between the three groups was conducted using a Kruskal–Wallis test in MATLAB.

### Odorant stimuli and delivery

Odorants (Sigma-Aldrich) were diluted from saturated vapor using a flow dilution olfactometer (Vucinić et al., 2006), a design which generates a constant headspace concentration throughout the trial. Ethyl tiglate, methyl valerate, isoamyl acetate 2-heptanone and an odorant mixture (methyl valerate, isoamyl acetate, and 2-heptanone mixed in equal proportions in liquid phase) were delivered between concentrations of 0.06% and 2% of saturated vapor. The odor stimuli used for the epifluorescence imaging experiments are listed in Table 1. In a subset of the experiments a photo-ionization detector (200B mini-PID, Aurora Scientific) confirmed the time course and relative concentrations delivered by the olfactometer. The photo-ionization measurements in Extended Data Figure 1-2 were taken while the sensor was placed in front of the olfactometer located in front of the animal’s nose in a subset of the experiments (which is the cause of the “sniff” pattern in the red trace in Extended Data Fig. 1-2*A*).

We confirmed that the olfactometer delivered repeatable odor pulses by comparing the amplitude of the photoionization detector signal in response to the first and third odor presentation on eight different recording days in response to different odors and concentrations. The amplitude of the third odor pulse changed minimally relative to the first odor pulse (1.3 ± 1.7% SEM) when averaging individual trials using different odorants and concentrations.

### Histology

Mice were euthanized with euthasol, and their brains were dissected and left in 4% paraformaldehyde for a minimum of 3 d. Olfactory bulbs were embedded in 3% agarose and cut on a vibratome in 50- to 75-μm-thick coronal sections. Sections were mounted and coverslipped with VECTASHIELD Mounting Medium with DAPI (Vector Labs, H-1500) or Propidium Iodide (Vector Labs, H-1300). Slides were examined using a Zeiss LSM-780 confocal microscope (Carl Zeiss Microsystems). Appropriate targeting of the sensors was confirmed by visual inspection.

The fluorescence for the images in Figure 1*A* and Extended Data Figure 1-3 were generated without additional amplification steps. Images were uniformly contrast-enhanced and sharpened, cropped and pseudo-colored using Zen Lite 2011 (Carl Zeiss Microsystems), Adobe Photoshop and Illustrator (Adobe Systems Inc.).

## Results

### Input-output transformation

#### Simultaneous measurements of input and output

Figure 1*A* shows an example histologic result in which olfactory sensory neurons were loaded with an organic calcium dye (Cal-590 dextran) via nasal infusion (Fig. 1*A*, left panel; Friedrich and Korsching, 1997; Wachowiak and Cohen, 2001; Fried et al., 2002; Storace and Cohen, 2017) in a transgenic mouse that expressed the GECI GCaMP6f in the mitral/tufted output cells (Fig. 1*A*, middle panel; Dana et al., 2014). The merged result (Fig. 1*A*, right panel) shows that the input and output processes overlap in the bulb glomeruli, and thus, measurements from the glomeruli will have activity signals from both the olfactory receptor neuron input and the mitral/tufted cell output.

Alternative imaging frames measured the fluorescence induced by two different excitation wavelengths and thus the two different activity sensors. Input and output activity measurements were made using a microscope with two LED light sources. The LEDs were synchronized with the camera so that only one LED illuminated the preparation in each camera frame (Extended Data Fig. 1-1*A*; Miyazaki and Ross, 2015; Miyazaki et al., 2018). Because the excitation spectra of the fluorophores used in these experiments had minimal overlap, there was minimal cross talk between the fluorophores. Strobing the LEDs did not noticeably impact the imaging signal-to-noise ratio. This can be seen by comparing measurements from trials using continuous illumination to those in which the LED was strobed (in a preparation that had only a single fluorophore, GCaMP6f). Odor-evoked signals were measured during trials in which the blue LED was on continuously, and in separate trials where the blue LED was only on during alternate camera frames (Extended Data Fig. 1-1*B*, top vs bottom). The signals from the continuous imaging trial were similar to those from the LED-On frames of the strobing trial, while the LED-Off frames had no detectable signal (Extended Data Fig. 1-1*C*, black trace). Thus, this approach allows effectively simultaneous measurements of the input-output transformation in a single trial.

#### Simultaneous wide-field input and output measurements from the same glomeruli

Odor-evoked responses were measured from the bulb input and output in response to repeated 3-s presentations of the same odor stimulus with a 6-s interstimulus interval in awake or anesthetized head-fixed mice. We confirmed that the olfactometer could deliver stable odor pulses using a photoionization detector (Extended Data Fig. 1-2).

Figure 1*B–D* shows measurements from a preparation in which the olfactory receptor neurons were labeled with the organic calcium dye Cal-590 dextran via nasal infusion in the Thy1-GCaMPf 5.11 transgenic line that expresses GCaMP6f in the mitral/tufted cells (Dana et al., 2014; Iwata et al., 2017). The image of the responses to each odor presentation (methyl valerate at 2% of saturated vapor) was visualized by performing a frame subtraction analysis (see Materials and Methods) for both the input and output (Wachowiak and Cohen, 2001; Fig. 1*B*, activity maps).

In this preparation, the input activity map evoked by the third odor presentation was very similar to the activity map evoked by the first odor presentation. This is visualized by scaling the third activity map at the same scale as used for the first presentation (Fig. 1*B*, top, input, left vs right panel). Thus, the input measurements were stable across odor repeats, exhibiting minimal adaptation. In contrast, the magnitude of the output response changed substantially across odor presentations. Applying the same scaling analysis to the output activity maps revealed reductions in signal amplitude in almost all of the activated glomeruli (Fig. 1*B*, bottom, output, left vs right panel).

Single trial fluorescence time course measurements from two activated glomeruli are shown in Figure 1*C*. The presence of a glomerular sized peak in the activity map indicates that a glomerulus is more strongly activated than the surround. This is evident by comparing the fluorescence time course measured from a glomerulus versus its surrounding area (Fig. 1*C*, left panel, color vs black traces). For the input measurements, the separation between the center and surround were similar for the three odor pulses (Fig. 1*C*, right panel). In contrast, the difference between the center and surround of the output signals became smaller (Fig. 1*C*, right panel, output). The results from these two glomeruli are consistent with the changes visualized in the input and output activity maps (Fig. 1*B*). The difference between the center and surround of activated input and output glomeruli was used to quantify the amount of adaptation. The amplitude of the bulb input declined much less than the output (Fig. 1*D*). For this preparation, the input declined to 0.96 ± 0.05, while the output declined to 0.68 ± 0.07 (*p* < 0.005; *N* = 16 glomeruli, output vs input).

While the Thy1-GCaMP6f 5.11 transgenic line selectively expresses GCaMP6f in mitral/tufted cells in the olfactory bulb, some expression also exists in the cortex (Dana et al., 2014). We were concerned that some of these cortical neurons could feedback to the olfactory bulb, which could introduce confounding optical signals. We repeated this experiment using the Tbx21-Cre mouse line, which expresses Cre recombinase exclusively in mitral/tufted cells (Mitsui et al., 2011). This line was then crossed to a transgenic GCaMP6f reporter mouse (Madisen et al., 2015). Histologic examination confirmed that the GCaMP6f expression was restricted to the mitral/tufted cells (Extended Data Fig. 1-3*A*). In these preparations, Cal-590 dextran was loaded into the olfactory receptor neurons using nasal infusion. The results from an example preparation are shown in Extended Data Figure 1-4, which has an identical display arrangement as that used in Figure 1*B–D*. On average the output declined more than the input (input: 0.87 ± 0.08; output: 0.49 ± 0.1; *p* = 0.06; *N* = 4 glomeruli, output vs input). Thus, the differences between input and output are unlikely to be explained by the choice of the transgenic mouse line.

#### Using other combinations of calcium sensors

Different optical indicators can vary in their signal-to-noise ratio, and calcium indicators have different affinities (Kd), Hill coefficients, and photobleaching properties (Tian et al., 2009; Akerboom et al., 2012; Chen et al., 2013; Sun et al., 2013; Badura et al., 2014; Dana et al., 2016). To control for these effects, we generated a transgenic mouse line in which we could record from the olfactory sensory neuron input using a protein-based sensor (GCaMP3). The Tbx21-Cre transgenic mouse line was crossed to a transgenic mouse in which GCaMP3 (Dewan et al., 2018) was expressed in the bulb input (OMP-GCaMP3 x Tbx21-Cre). The mitral/tufted cell output was targeted with red calcium sensors, either jRCaMP1a or jRGECO1a (Dana et al., 2016) using a Cre-dependent AAV. Histology demonstrating targeting of the sensors in this double transgenic mouse line is shown in Extended Data Figure 1-3. jRCaMP1a was used for the preparation in Extended Data Figure 1-5 where the input and output declined to 0.97 ± 0.06 and 0.85 ± 0.07 (*N* = 23 glomeruli, *p* = 0.14, output vs input), respectively. jRGECO1a was used for the preparation in Extended Data Figure 1-6. However, input and output measurements were performed in alternate imaging trials because blue light activates slow and sustained rises in jRGECO1a fluorescence, which precluded simultaneous measurements (Extended Data Fig. 1-7). Regardless, the results were consistent with the other preparations where the normalized response to the third odor presentation for input and output was 1.0 ± 0.04 and 0.92 ± 0.1 (*N* = 7 glomeruli, *p* = 0.16, output vs input), respectively. Thus, we measured effects in the same direction regardless of whether the input was recorded using an organic dye or a protein-based calcium indicator.

Next, we assessed whether output adaptation was dependent on anesthetic state by carrying out input-output measurements in three anesthetized mice (olfactory receptor neurons labeled with Cal-590 in Thy1-GCaMP6f 5.11 mice; Fig. 2 and Table 1, preparations 14–16). A total of 94 glomeruli-odor pairs (34, 39, and 21 per preparation) were recorded in response to three different odors across four concentrations (between 0.12% and 2% of saturated vapor). Consistent with the prior reports, odors evoked more diffuse output signals in anesthetized mice in comparison to measurements from awake animals (Blauvelt et al., 2013; Wachowiak et al., 2013). In any case, the output still adapted more on average than the input after applying the center-surround subtraction analysis (input: 0.78 ± 0.12; output: 0.64 ± 0.13; *N* = 3 preparations, not statistically significant). Comparisons of input and output measurements in awake (*N* = 13 preparations) versus anesthetized (*N* = 3 preparations) mice were not significant (input: rank-sum = 123; *p* = 0.11; output: rank-sum = 118, *p* = 0.36). Thus, the ability of the olfactory bulb to contribute to sensory adaptation does not require wakefulness.

We determined whether different bleaching kinetics of the sensors could explain the result by measuring the change in the absolute fluorescence intensity at the beginning of the imaging trial and immediately before the delivery of the third odor pulse. The different sensors had similarly small bleaching effects: Cal-590 (0.98 ± 0.01; *N* = 6), GCaMP3 (0.94 ± 0.01; *N* = 4), GCaMP6f (0.97 ± 0.01, *N* = 6), jRGECO1a (0.96 ± 0.01, *N* = 2), and jRCaMP (0.95, *N* = 1; values are the fluorescent intensity of the third odor pulse baseline normalized to the first odor pulse baseline). On average the input and output measurements exhibited relatively small and similar decreases (input: 0.96 ± 0.01; output: 0.96 ± 0.01; *N* = 10). Thus, bleaching similarly affected both input and output, and by a magnitude much smaller than the effects of adaptation on the output.

#### Summary of same glomeruli input-output comparisons

We compared input and output measurements in 16 preparations (13 awake, 3 anesthetized) using 2 different input sensors (Cal-590: 11 preparations; GCaMP3: 5 preparations) and three different output sensors (GcaMP6f: 11 preparations, jRCaMP1a: 2 preparations; jRGECO1a: 3 preparations; Fig. 2*A*). The sensors, odorants and odorant concentrations used in these measurements are indicated in Table 1. Not all glomeruli exhibited the same behavior in each preparation (e.g., the output declined less than the input in some glomeruli), and not all preparations had within comparisons that were statistically significant. However, all but one of the experiments had means in the same direction (Fig. 2*A*). The average normalized response to the third odor preparation was averaged across all activated glomeruli per preparation (15 ± 2.6; between 1 and 39 glomeruli per preparation). The input and output declined significantly from baseline conditions to 0.88 ± 0.03 and 0.71 ± 0.04, respectively (input: *p* < 0.005; output: *p* < 0.001; Wilcoxon signed-rank test). However, the output declined significantly more than the input (*p* < 0.005; Wilcoxon rank-sum test; Fig. 2*B*). Statistical and experimental details for the individual preparations and the population are included in Table 1. Thus, the mitral/tufted cells adapt significantly more than olfactory receptor neurons; the olfactory bulb contributes to adaptation.

Moreover, preliminary results measuring olfactory receptor neuron activity using the Genetically Encoded Voltage Indicator, ArcLight showed comparable adaptation responses as those measured here using calcium sensors (Platisa et al., 2020). This strengthens the conclusion that the choice of optical sensor is unlikely to explain the measured differences between input and output.

#### Measuring input and output using the same sensor in different preparations

We also controlled for the effects of sensor differences by using the same protein sensor to record from both the input and output, albeit in different preparations. In these experiments, GCaMP6f was used to measure the olfactory bulb input in an OMP-Cre transgenic mouse that had been crossed to a GCaMP6f reporter mouse (Madisen et al., 2015). We compared these input results to the preparations in Table 1 in which GCaMP6f had been used to measure the mitral/tufted output. The mitral/tufted cell output declined significantly more than the olfactory receptor neuron input when both were measured using GCaMP6f (input: *N* = 4 preparations, 0.86 ± 0.03; output: *N* = 11 preparations, 0.65 ± 0.04; *p* < 0.01 with Wilcoxon rank-sum test).

We also determined whether the magnitude of olfactory receptor neuron adaptation was different when measured using either an organic calcium dye or a protein sensor by comparing input adaptation measured with different sensors. The effect of repeated stimulation on olfactory receptor neurons lead to a similarly modest effect on response amplitude regardless of the sensor (Cal-590: 0.86 ± 0.03; GCaMP3: 0.93 ± 0.05; GCaMP6f: 0.86 ± 0.03; χ^2^ = 1.7, *p* = 0.43, Kruskal–Wallis test). Thus, input measurements conducted using an organic calcium dye and two different protein-based GECIs gave similar results.

#### Measuring input and output using two-photon imaging in separate anesthetized preparations

The optical signals from wide-field imaging can be influenced by fluorescence originating from above and below the objective focal plane. Accordingly, we made similar measurements using two-photon microscopy, which has a much smaller depth-of-field (Denk et al., 1990), to test the possibility that our results could be explained by out of focus signals. In these experiments, input and output were measured in different, anesthetized preparations. The input signals were measured in transgenic mice expressing either GCaMP3 (OMP-GCaMP3, *N* = 5 preparations) or GCaMP6f (OMP-Cre x Flex-GCaMP6f, *N* = 1 preparation). Output signals were measured in transgenic mice expressing either GCaMP6f (Thy1-GCaMP6f 5.11, *N* = 5 preparations; Tbx21-Cre x Flex-GCaMP6f, *N* = 3 preparations) or jRGECO1a (Tbx21-Cre injected with a Cre-dependent jRGECO1a-expressing AAV, *N* = 1 preparation).

Odor responses were measured to different odors (methyl valerate, isoamyl acetate, ethyl tiglate, and 2-heptanone) between 0.4% and 2% of saturated vapor, and the same glomeruli were tested across multiple stimulus conditions when possible. Odor responses were measured in 103 individual input glomeruli (between 7 and 31 glomeruli per preparation), resulting in a total of 202 odor-glomerulus input measurements. Odor responses were measured in 269 individual output glomeruli (between 9 and 56 glomeruli per preparation), resulting in a total of 494 odor-glomerulus measurements. The activity maps and fluorescence traces from two different preparations in which only the input or only the output was measured are shown in Figure 3. The activity maps are scaled in the same manner as those in Figure 1. Consistent with the wide-field fluorescence imaging experiments, the input measurements were relatively similar across odor repeats, while the output declined (Fig. 3*A*, see reduced hot colors for most output glomeruli). Fluorescence traces from some of the activated glomeruli are shown to demonstrate the differences (Fig. 3*B*). The mean normalized response to the fourth odor presentation for the input preparation was 0.85 ± 0.03 (*N* = 25 odor-glomeruli pairs), while the mean of the output preparation was 0.6 ± 0.04 (*N* = 77 odor-glomeruli pairs; Fig. 3*C*). Similar results were obtained across a population of preparations where the mean normalized response to the third odor presentation for the input was 0.83 ± 0.11 (*N* = 6 preparations), while the mean of the output preparations was 0.66 ± 0.07 (*N* = 9 preparations; *p* < 0.005 using a Wilcoxon rank-sum test; Fig. 3*D*). Thus, the two-photon measurements gave results similar to those in wide-field measurements. Overall, measurements from input and output using different combinations of sensors in awake and anesthetized mice, using wide-field and two-photon fluorescence imaging, show that the olfactory bulb contributes to odor adaptation with the odorant presentation protocol we used. The bulb output adapts substantially more than its input.

#### Unusual glomerular response types measured using two-photon and wide-field imaging

##### Input (two-photon)

The majority of input glomeruli exhibited increases with different onset kinetics that were consistent with previous reports (Wachowiak and Cohen, 2001; Spors et al., 2006). Only two glomeruli had suppressed responses (2/103 individual glomeruli; data not shown).

##### Output (two-photon)

The majority of output glomerular measurements responded quickly to the odorant and returned to baseline following the removal of the odor (Extended Data Fig. 3-1*A*, 438/500 glomerular measurements). The remaining measurements (62/500) exhibited more complex response dynamics not seen in the input measurements. Single trial recordings of different response types are included in Extended Data Figure 1-1*B–G*. Some glomeruli exhibited both an on and off response (Extended Data Fig. 3-1*B*,*C*; 28/500). Others exhibited a polarity change to repeated odor presentations. These glomeruli typically had a slow increase in fluorescence to the first odor presentation, and suppression to subsequent odor presentations (Extended Data Fig. 3-1*D*; 18/500). Other response types included an off response (Extended Data Fig. 3-1*E*; 2/500), an off-activity increase followed by suppression (Extended Data Fig. 3-1*F*; 2/500) and glomeruli whose activity was suppressed by the odor (Extended Data Fig. 3-1*G*; 9/500). Note that each trace presented in Extended Data Figure 3-1*B–G* was recorded in a trial that had a response in another glomerulus consistent with Extended Data Figure 3-1*A* (i.e., a ON response). Many of these response types are consistent with previous reports using electrode measurements and glomerular recordings from mitral/tufted cells (Sirotin et al., 2015; Economo et al., 2016; Bolding and Franks, 2018; Parabucki et al., 2019).

##### Output (wide-field)

In addition, a small fraction of our wide-field epifluorescence mitral/tufted recordings also exhibited unusual response properties (Extended Data Fig. 3-1*I–K*). The presence of glomeruli that switch polarities (Extended Data Fig. 3-1*D*,*J*) and those with suppressed responses to all the odor presentations (Extended Data Fig. 3-1*K*) suggest that glomerulus specific inhibition occurs. Because none of the input glomeruli had similar characteristics, these effects appear to be generated by processing within the olfactory bulb. In some preparations there were wide-spread areas with suppressed responses (Extended Data Fig. 3-2). The suppressed responses often did not change in response to repeated odor presentations, strongly suggesting that broad increases in suppressed signals cannot explain the output adaptation.

## Discussion

Here, we provide the first simultaneous comparison between the olfactory sensory neuron input and the mitral/tufted cell outputs from many glomeruli in the same preparation in response to repeated odorant presentations. Although prior studies have shown adaptation occurring in both olfactory receptor neurons and mitral/tufted cells (Leinders-Zufall et al., 1998; Reisert and Matthews, 1999, 2001a; Wilson, 2000b; Verhagen et al., 2007; Ghatpande and Reisert, 2011; Ogg et al., 2015), distinguishing adaptation that occurs as a result of bulb processing from that inherited from the periphery requires recording from both cell populations simultaneously.

Measurements from the olfactory receptor neuron input were relatively stable in response to repeated odor presentations, while the mitral/tufted signal declined significantly more. This was true for both wide-field and two-photon measurements. The results demonstrate that the olfactory sensory neurons provide a more faithful representation of the sensory environment, while processing in the olfactory bulb reduces the output signal as a result of repeated presentations. This function is likely to be critical for identifying novel or important odor stimuli from an ongoing odor background.

### Methodological considerations

#### Near-simultaneous measurements of input and output using strobing LEDs

Because adaptation may be experience-dependent, we wanted a way to perform the input and output measurements simultaneously. Multiplexing the LED excitation was preferable to using a dual bandpass filter to simultaneously excite both fluorophores since many fluorophores have broad excitation and emission spectra, which could introduce cross talk between the channels. We concluded that the noise introduced by the strobing approach was negligible.

#### Sensor differences

Different optical indicators can vary in their signal-to-noise ratio, and calcium indicators exhibit different calcium affinities (Kd), Hill coefficients and photobleaching properties (Tian et al., 2009; Akerboom et al., 2012; Chen et al., 2013; Sun et al., 2013; Badura et al., 2014; Dana et al., 2016). We attempted to address possible effects of the sensors we used by using three strategies. First, we conducted input and output measurements using different combinations of optical sensors with different biophysical properties (Table 1). Second, by using the same sensor (GCaMP6f) in different preparations. Third, by demonstrating that the input measurements were similar regardless of the sensor used. Furthermore, olfactory receptor neuron adaptation measured using a voltage indicator gave similar results (Platisa et al., 2020). Thus, it is unlikely that the reported differences between input and output are artifactual. Furthermore, our recent comparisons of input and output for a different olfactory perception (Storace and Cohen, 2017; Storace et al., 2019) used an even larger number of different calcium and voltage indicators, all of which yielded similar results. That said, one caveat is that these experiments do not address the possibility that the axon terminals and dendrites could have different kinetic properties which could affect our conclusion.

#### The relationship between calcium and neurotransmitter release

A concern is that calcium or voltage measurements might not directly reflect synaptic release in the nerve terminals of olfactory receptor neurons, a result described in the *Drosophila* olfactory system (Kazama and Wilson, 2008; Martelli and Fiala, 2019). Calcium influx and synaptic vesicle release are significantly correlated when using an odor paradigm with intermittent odor stimulation (Bozza et al., 2004), although this might not be true under the paradigm we used to induce adaptation. Indeed, *in vitro* measurements of olfactory receptor neuron synaptic vesicle release exhibited stronger paired-pulse depression than measured using calcium (Wachowiak et al., 2005). However, other *in vitro* studies that examined the effects of olfactory nerve layer stimulation on postsynaptic targets reported more rapid recovery (Aroniadou-Anderjaska et al., 2000; Murphy et al., 2004). Clearly, olfactory receptor neurons exhibit synaptic depression under some conditions, which would be transmitted to the mitral/tufted cells. The relative proportion of mitral/tufted cell adaptation that can be attributed to depression at the olfactory receptor neuron synapse needs to be addressed in future work using either sensors of synaptic vesicle release (Bozza et al., 2004; Wachowiak et al., 2005) or glutamate targeted to postsynaptic neurons (Marvin et al., 2013, 2018; Moran et al., 2021).

Another concern is the relationship of calcium measurements in the apical dendrites of mitral/tufted cells to spiking activity in the soma, since synaptic potentials can also evoke increases in calcium (Charpak et al., 2001). However, these subthreshold signals evoke much smaller increases in calcium than action potentials (Charpak et al., 2001; Zhou et al., 2006). In addition, because action potentials propagate actively over the entire length of the primary dendrite (Debarbieux et al., 2003; Djurisic et al., 2004; Zhou et al., 2006), the calcium signal in the dendritic tufts very likely mainly reflects action potential activity in the mitral/tufted output neurons.

#### Use of a center-surround subtraction to correct for diffuse fluorescence in one-photon experiments

Diffuse fluorescence has been described in epifluorescence imaging experiments from both olfactory receptor neuron glomeruli (Wachowiak and Cohen, 2001; Verhagen et al., 2007) and mitral/tufted cell glomeruli (Fletcher et al., 2009; Blauvelt et al., 2013). Diffuse signal likely reflects some combination of out-of-focus signal and hemodynamics, and these prior studies have used a similar background-subtraction analysis to correct for the diffuse signal. Thus, there is precedent from prior studies to use a similar analysis applied to both olfactory receptor neurons and mitral/tufted cells. Moreover, two results support the validity of this analysis. First, the results of the center-surround analysis were consistent with our frame subtraction analysis. Second, we obtained similar results using two-photon imaging, which greatly increased the *z*-axis resolution and thus restricted the measurement to the glomerular layer. Thus, we conclude that the center-surround analysis is a reasonable method for correcting diffuse fluorescence.

### Comparison with previous reports

Odorant receptors are known to exhibit adaptation to continuous odor streams or paired pulse stimulation (Kurahashi and Menini, 1997; Reisert and Matthews, 1999, 2001b; Zufall and Leinders-Zufall, 2000; Lecoq et al., 2009; Ghatpande and Reisert, 2011). However, intact olfactory receptor neurons stimulated with paired pulses of odorants exhibited substantial recovery of the initial response with a 6-s delay between pulses (Kurahashi and Menini, 1997; Zufall and Leinders-Zufall, 2000; Murphy et al., 2004; Wachowiak et al., 2005). We used an intertrial interval of 6 s, which allowed time for the input signal to partially recover from any adaptation that occurred from the preceding odor pulse.

Although our study indicates that mitral/tufted cells adapt to odor stimuli, there are prior reports in which they exhibit relatively sustained responses to a constant or repeated odor stimulus (Sobel and Tank, 1993; Wilson, 1998; Cang and Isaacson, 2003; Kadohisa and Wilson, 2006). These differences could reflect diversity of mitral/tufted cell adaptation, as well as the odor stimulation paradigm. Indeed, Figure 2 demonstrates that the average of the mitral/tufted cells in different glomeruli exhibit quite different degrees of adaptation.

The glomerular mitral/tufted cell outputs occasionally exhibited activity suppression in response to the initial odor presentation in awake animals (Extended Data Figs. 3-1, 3-2). Suppressed responses were not evident in prior reports of glomerular measurements from mitral/tufted cells in anesthetized mice using wide-field imaging (Fletcher et al., 2009; Ogg et al., 2015; Storace and Cohen, 2017), although they were reported in anesthetized mice when measured using two-photon imaging (Economo et al., 2016).

### Possible mechanisms and future work

In earlier experiments, we found that the olfactory bulb transforms an input which is a confound of odorant quality and concentration into an output which is relatively concentration invariant (Storace and Cohen, 2017). Thus, the olfactory bulb participates in at least two computations simultaneously that may be related to perception. Indeed, despite its position as the first stage of olfactory information processing, the olfactory bulb has also been shown to participate in additional diverse functions including metabolic sensitivity (Fadool et al., 2011; Thiebaud et al., 2014; Riera et al., 2017), learning (Kass et al., 2013), and context-dependent processing (Doucette et al., 2011; Nunez-Parra et al., 2014; Li et al., 2017; Koldaeva et al., 2019; Wang et al., 2019).

Because the computations that occur in the olfactory bulb about concentration invariance and adaptation occur on a second (or subsecond) time scale, it suggests that the mechanisms are circuit based and do not involve slower processes such as changes in gene expression. However, these computations could presumably involve local circuits as well as processing across multiple brain areas.

The role of each of the large number of individual olfactory bulb interneuron types (Parrish-Aungst et al., 2007) need to be examined. Indeed processing within the olfactory bulb network has been shown to be important for both concentration invariance (Banerjee et al., 2015)} as well as for adaptation measured in mitral/tufted cells (Margrie et al., 2001; Dietz and Murthy, 2005). NMDA receptor modulation has been shown to play a role in olfactory bulb adaptation over much longer timescales (Chaudhury et al., 2010). Moreover, mitral/tufted cells send axons to twelve brain areas (Davis and Macrides, 1981; Igarashi et al., 2012), and almost all of those brain areas send feedback projections back to the olfactory bulb (Shipley and Ennis, 1996; Mandairon et al., 2014; In 't Zandt et al., 2019; Schneider et al., 2020). This centrifugal feedback appears to play a clear role in shaping the long-term olfactory bulb output as evidenced by experiments showing the impact of modulation of the locus coeruleus on adaptation over long timescales (Ogg et al., 2018), as well as the finding that mitral/tufted cell activity is experience and context dependent (Doucette et al., 2011; Wang et al., 2019). These data indicate that in the long-term mitral/tufted cell activity is shaped by sensory experience transmitted from higher brain centers, although future experiments are needed to study the role of feedback in the short-term perceptual calculations conducted by the bulb.

The results presented here indicate that processing within the bulb can significantly transform a relatively stable olfactory sensory neuron input. In principle, a decreased mitral/tufted output would be useful for higher brain regions to know whether the olfactory input is novel versus part of a stable background. Future studies need to determine how long it takes for the mitral/tufted cells to recover. Is the adaptation odorant specific? Is the degree of adaptation altered by learned associations which are known to influence mitral/tufted cell activity (Wilson and Sullivan, 1992; Doucette et al., 2011; Nunez-Parra et al., 2014; Ross and Fletcher, 2018)?

Our results taken together with other recent work has shown that mitral/tufted cells are influenced by stimulus history (Vinograd et al., 2017; Parabucki et al., 2019), However, aside from an attenuated response, the functional transformation described here remains unclear. Future studies are needed to define whether this adaptation allows neurons to maximize their dynamic range, a function that exists in other sensory systems (Barlow, 1961; Fairhall et al., 2001; Wark et al., 2007; Whitmire and Stanley, 2016).

In conclusion, it is remarkable that these olfactory perceptual calculations are conducted in the very first processing stage of the mammalian olfactory pathway. In other sensory systems some perceptual responses only appear after processing by multiple brain regions (Van Essen et al., 1992; their Fig. 2). The finding that the olfactory bulb is carrying out two computations simultaneously has important implications for interpreting the synaptic connectivity in the olfactory bulb. Any given synaptic connection could be part of one, or the other, or both of the perceptual computations.

A well-known example of multiple computations in one brain region is V1 in visual cortex (Hubel and Wiesel, 1962; Augustinaite and Kuhn, 2020). But similar results have also been obtained in molluscan (Wu et al., 1994) and annelid (Briggman et al., 2005) nervous systems where individual neurons were shown to participate in more than one behavior. It seems likely that each brain region in all complex nervous systems will be similarly computing more than one output. This might result in the optimal use of a limited number of neurons as well as using an architecture that can more easily coordinate different behaviors.

## Synthesis

Reviewing Editor: Arianna Maffei, SUNY Stony Brook Decisions are customarily a result of the Reviewing Editor and the peer reviewers coming together and discussing their recommendations until a consensus is reached. When revisions are invited, a fact-based synthesis statement explaining their decision and outlining what is needed to prepare a revision will be listed below. The following reviewer(s) agreed to reveal their identity: NONE. Note: If this manuscript was transferred from JNeurosci and a decision was made to accept the manuscript without peer review, a brief statement to this effect will instead be what is listed below.

The manuscript has been transferred from J. Neuroscience. The authors have satisfactorily addressed the concerns of the reviewers.
