# Supplementary figures and images for: The Mammalian Olfactory Bulb Contributes to the Adaptation of Odor Responses: A Second Perceptual Computation Carried Out by the Bulb
Source: eNeuro. 2021 Sep 23;8(5):ENEURO.0322-21.2021. doi: 10.1523/ENEURO.0322-21.2021 (PMC8474650; doi:10.1523/ENEURO.0322-21.2021)

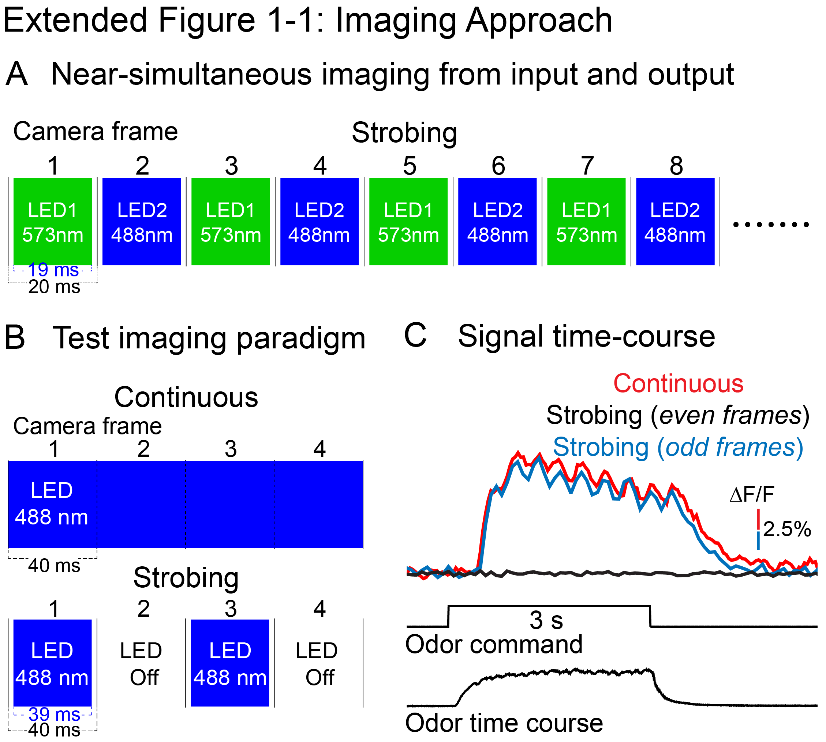

Supplement: Extended Data Figure 1-1 — A, Imaging paradigm for simultaneous measurements from the two fluorophores. B, C, Multiplexing LEDs does not notably impact the signal-to-noise ratio. In one experiment, GCaMP6f was imaged continuously (B, top), or was multiplexed so that alternate frames were blank (C, bottom). C, Odor-evoked fluorescence signals measured from the illuminated frames were not distinguishable from the continuous recording, and no signal was detectable in the LED-OFF camera frames (black trace). Similar results were obtained in two other preparations. Download Figure 1-1, TIF file. [file enu-eN-NWR-0322-21-s01.tif]

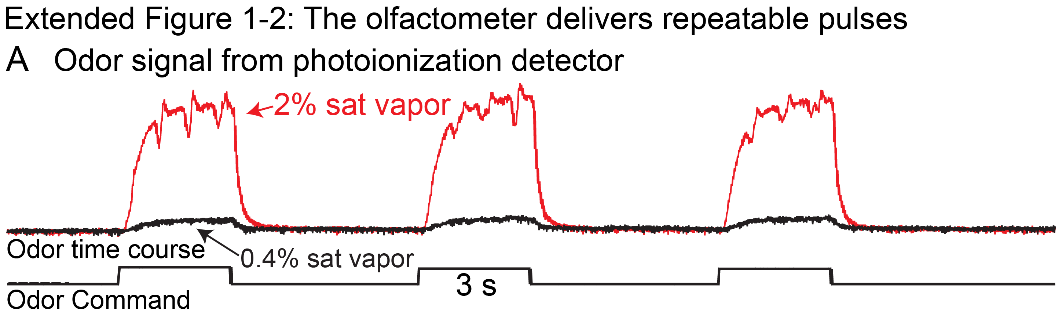

Supplement: Extended Data Figure 1-2 — The olfactometer delivers repeatable odor pulses. A photoionization detector was used to measure the output of the olfactometer across three odor repeats at two different concentrations. Similar recordings were taken across eight experiments on different days. Download Figure 1-2, TIF file. [file enu-eN-NWR-0322-21-s02.tif]

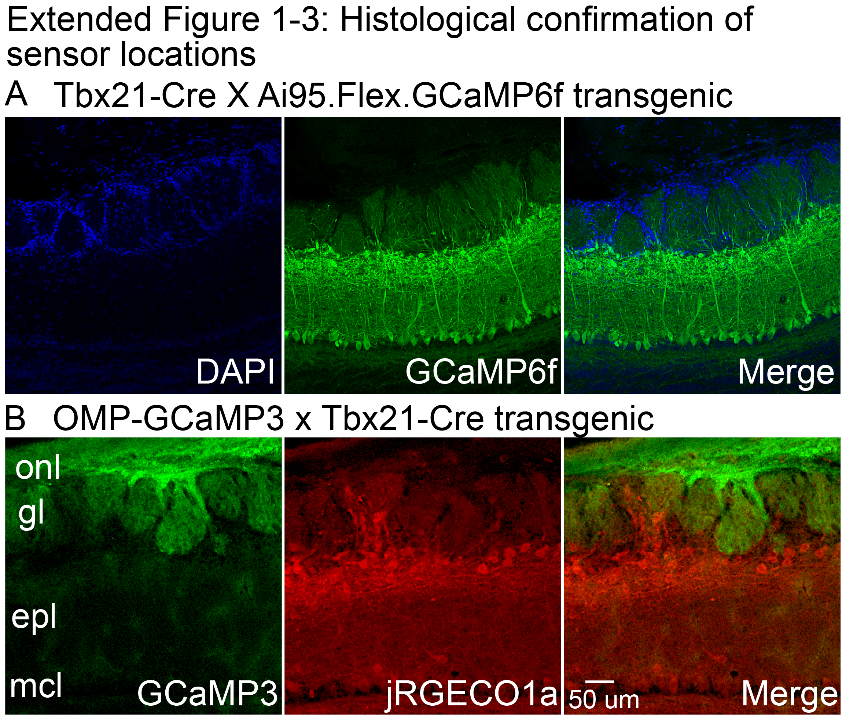

Supplement: Extended Data Figure 1-3 — Histology from three different transgenic mice showing expression of the sensors in the expected locations. A, Tbx21-Cre x Ai95.Flex.GCaMP6f transgenic mouse resulted in mitral/tufted cell-specific expression (middle panel). B, OMP-GCaMP3 x Tbx21-Cre transgenic mouse. GCaMP3 is targeted to the bulb input (left panel). jRGECO1a was expressed in the mitral/tufted cells using a cre-dependent AAV (middle panel). Download Figure 1-3, TIF file. [file enu-eN-NWR-0322-21-s03.tif]

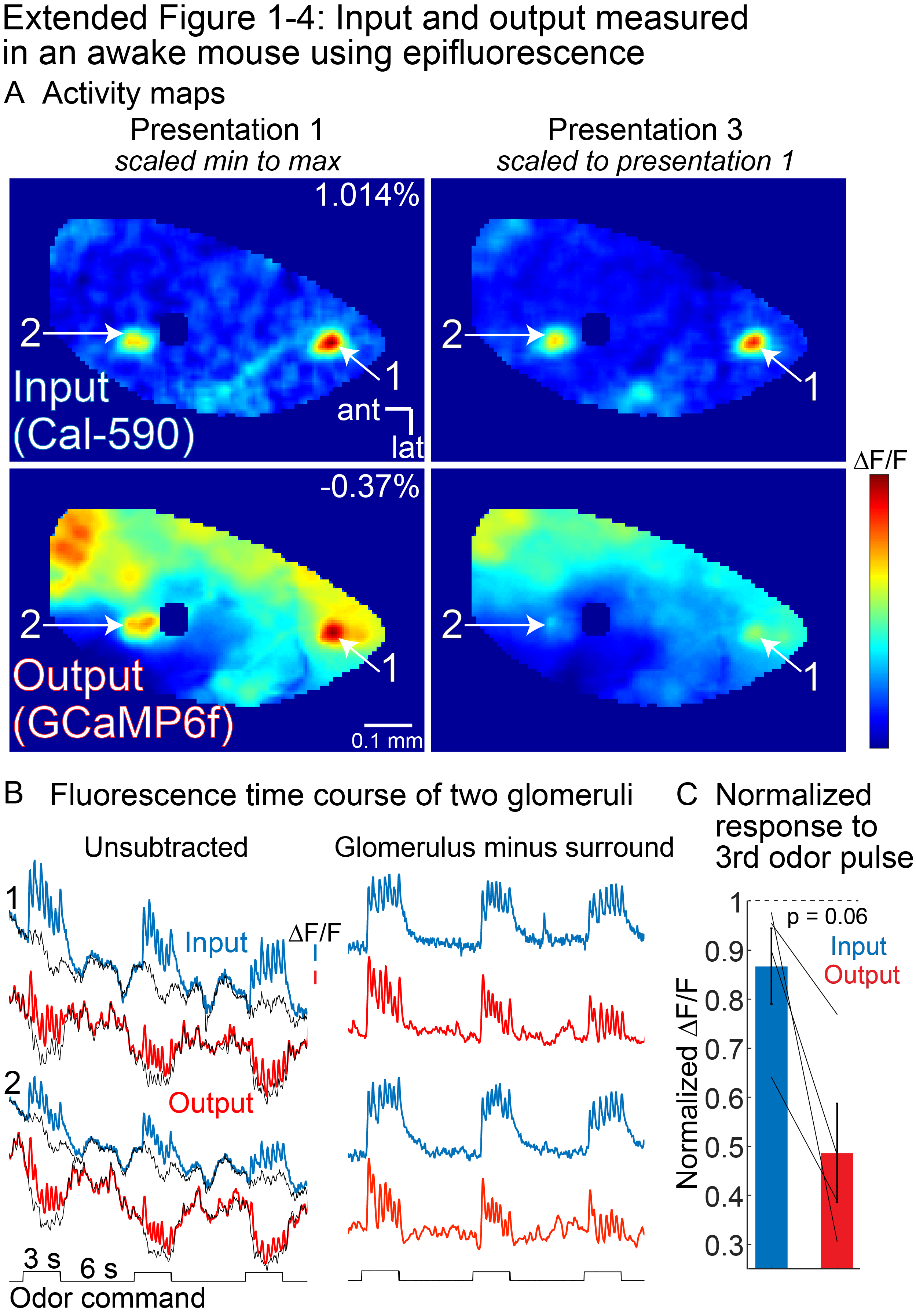

Supplement: Extended Data Figure 1-4 — The bulb output adapts more than the input in response to repeated odor stimulation in a Tbx21-Cre x Ai95.Flex.GCaMP6f transgenic mouse that had Cal-590 loaded into its OSNs. The data display arrangement and legend are otherwise identical to Figure 2. The odor used was isoamyl acetate at 2% of saturated vapor. The input and output scale bars in panel B indicate 0.5% and 2% ΔF/F, respectively. Download Figure 1-4, TIF file. [file enu-eN-NWR-0322-21-s04.tif]

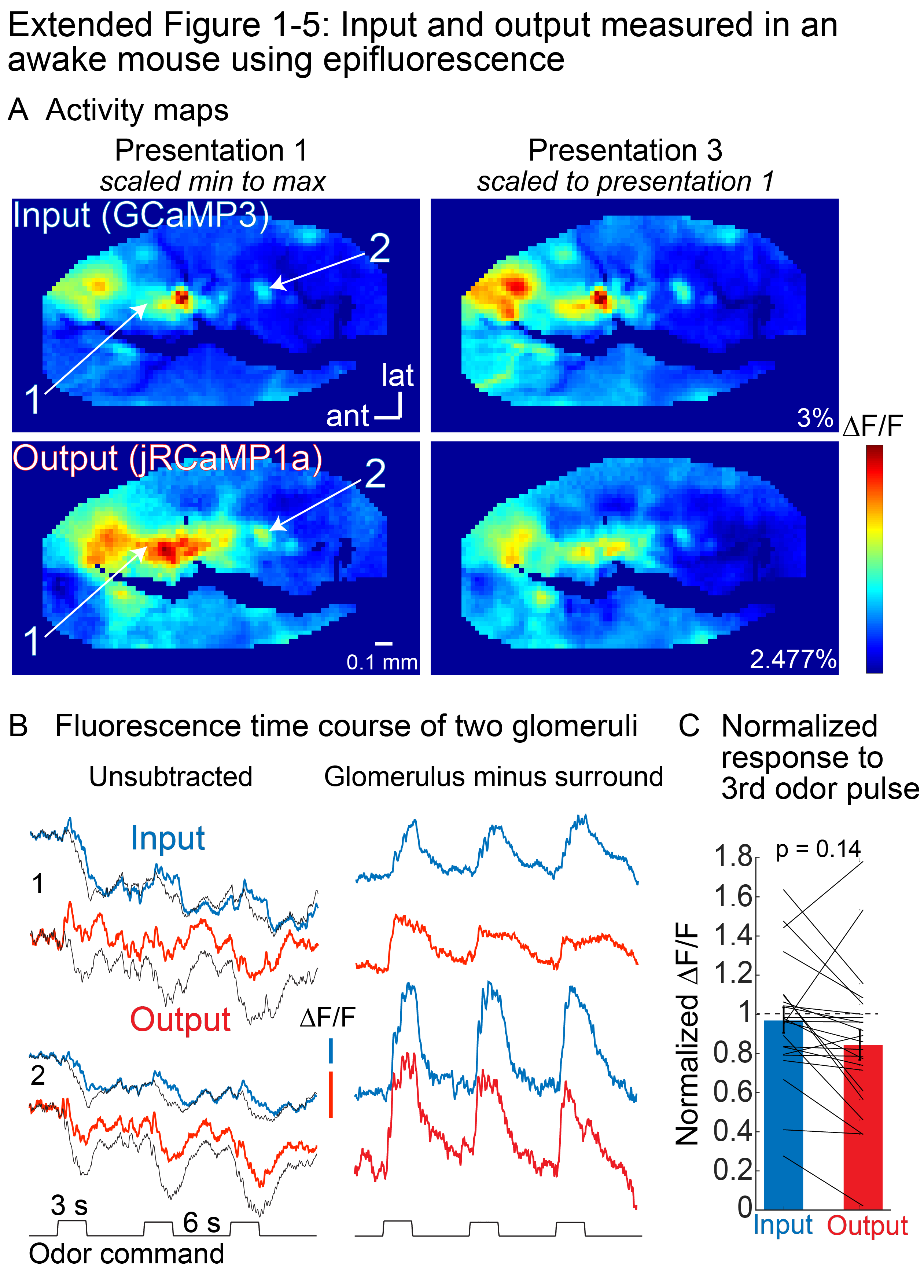

Supplement: Extended Data Figure 1-5 — The bulb output adapts more than the input in response to repeated odor stimulation in an OMP-GCaMP3 x Tbx21-Cre transgenic mouse that was injected with a cre-dependent AAV-expressing jRCaMP1a. The data display arrangement and legend are otherwise identical to Figure 2. The odor used was methyl valerate at 2% of saturated vapor. The input and output scale bars in panel B indicate 1.8% and 3% ΔF/F, respectively. Download Figure 1-5, TIF file. [file enu-eN-NWR-0322-21-s05.tif]

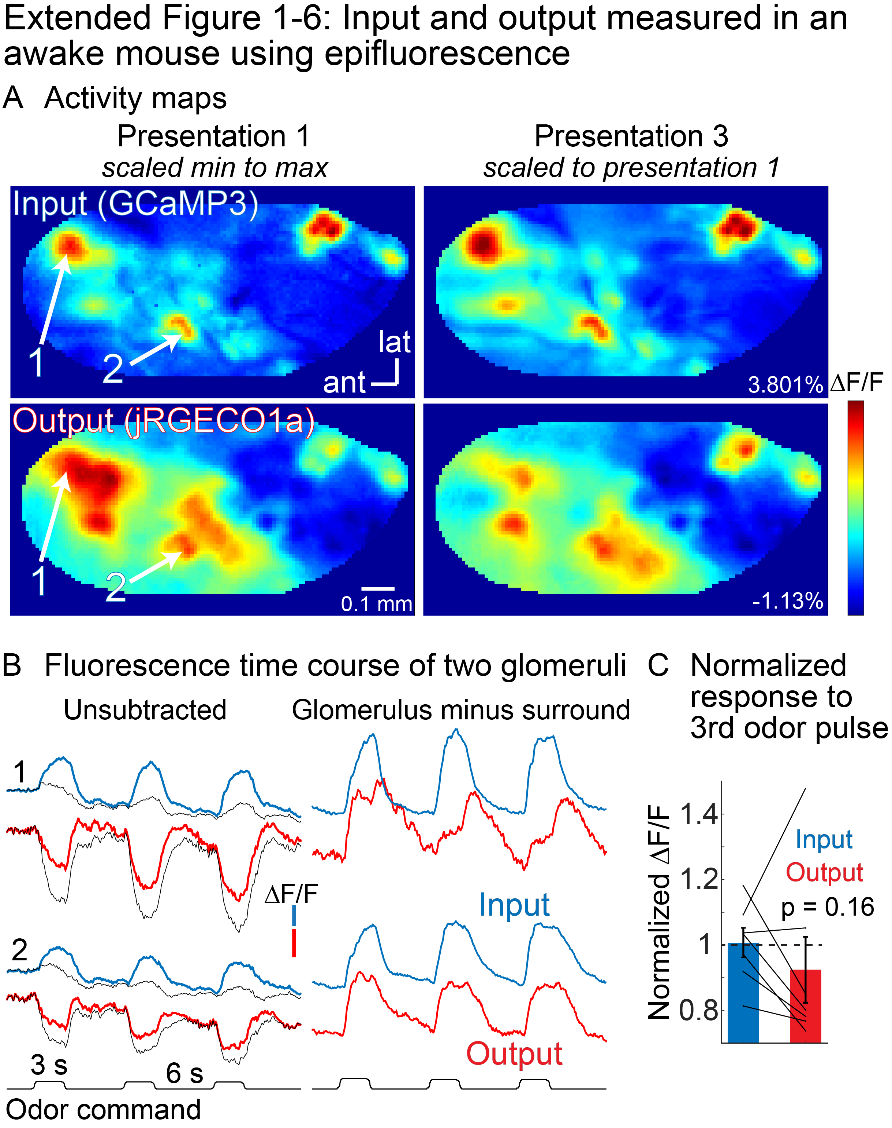

Supplement: Extended Data Figure 1-6 — The bulb output adapts more than the input in response to repeated odor stimulation in an OMP-GCaMP3 x Tbx21-Cre transgenic mouse that was injected with a cre-dependent AAV-expressing jRGECO1a. The data display arrangement and legend are otherwise identical to Figure 2. The odor used was methyl valerate at 2% of saturated vapor. The traces in panel B are from aligned averages. The input and output scale bars in panel B indicate 2% and 2% ΔF/F, respectively. Download Figure 1-6, TIF file. [file enu-eN-NWR-0322-21-s06.tif]

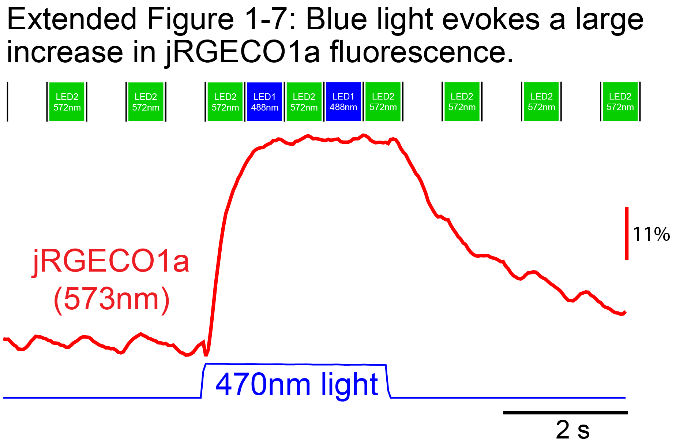

Supplement: Extended Data Figure 1-7 — Blue light evokes a large increase in jRGECO1a fluorescence emission in the absence of odor presentation. The green LED (573 nm) illuminated the preparation every other camera frame. No response is present while alternate frames are blank. The introduction of blue (470 nm) light on the alternate camera frames causes a very large, slow increase in jRGECO1a fluorescence emission that even more slowly returned to baseline after the blue LED was removed. Download Figure 1-7, TIF file. [file enu-eN-NWR-0322-21-s07.tif]

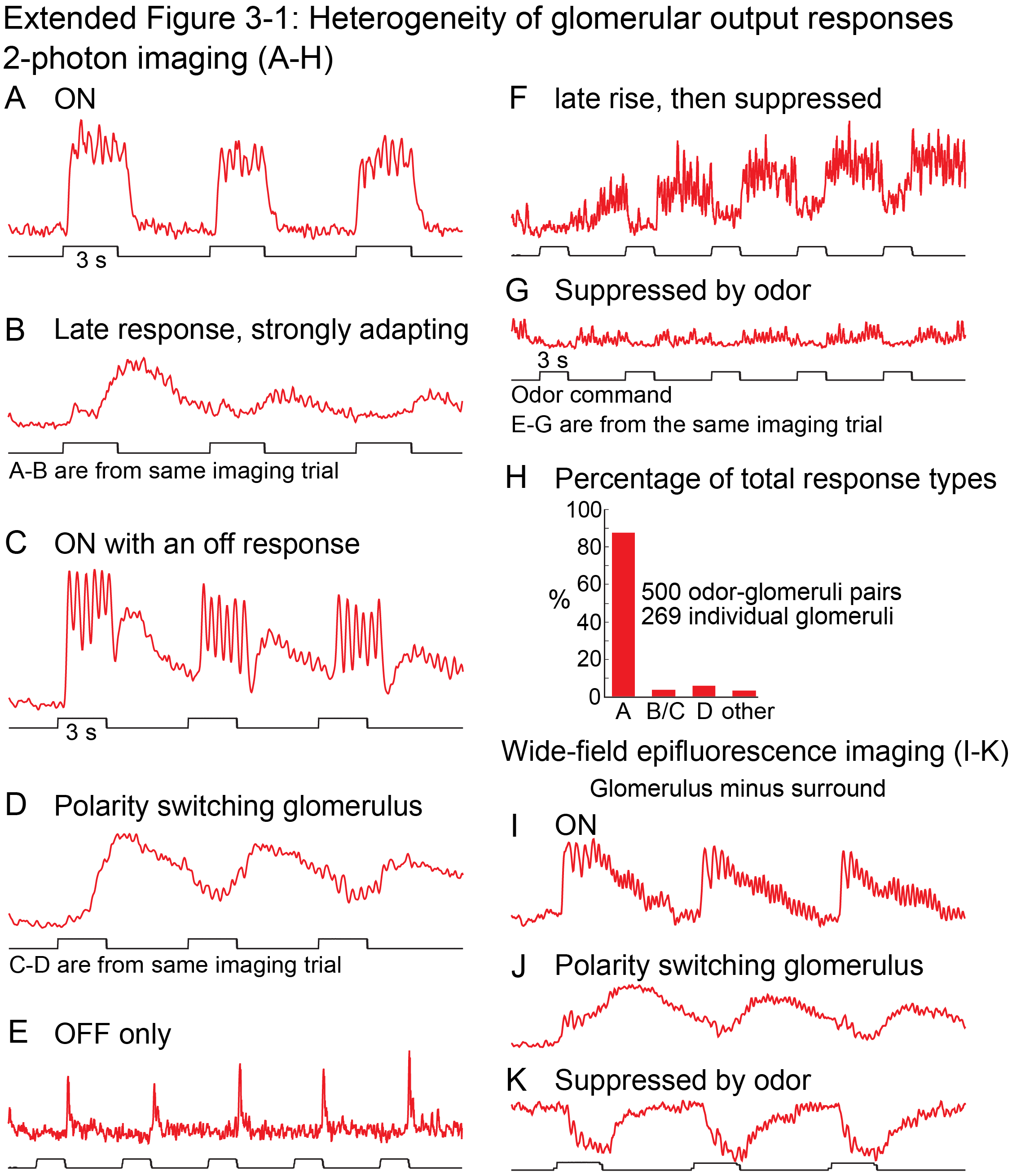

Supplement: Extended Data Figure 3-1 — Heterogeneity of glomerular output responses measured using two-photon and wide-field epifluorescence imaging. The glomerular mitral/tufted cell output exhibited diverse response types not seen in the olfactory sensory neuron input. A–H, Two-photon imaging: traces are from single trial measurements. The traces in A, B; C, D; and E, F are different glomeruli from the same imaging trial, but in three different preparations. H, Percentage response types across all two-photon output measurements. I–K, Wide-field epifluorescence imaging: glomerulus J exhibits a slow rise, and early suppression to the later odor pulses (similar to B, D). Glomerulus K exhibits a suppressed response to the odor (similar to F, G). The traces in I–K data are from the preparation shown in Figure 1. However, qualitatively similar observations were made in multiple preparations. Download Figure 3-1, TIF file. [file enu-eN-NWR-0322-21-s08.tif]

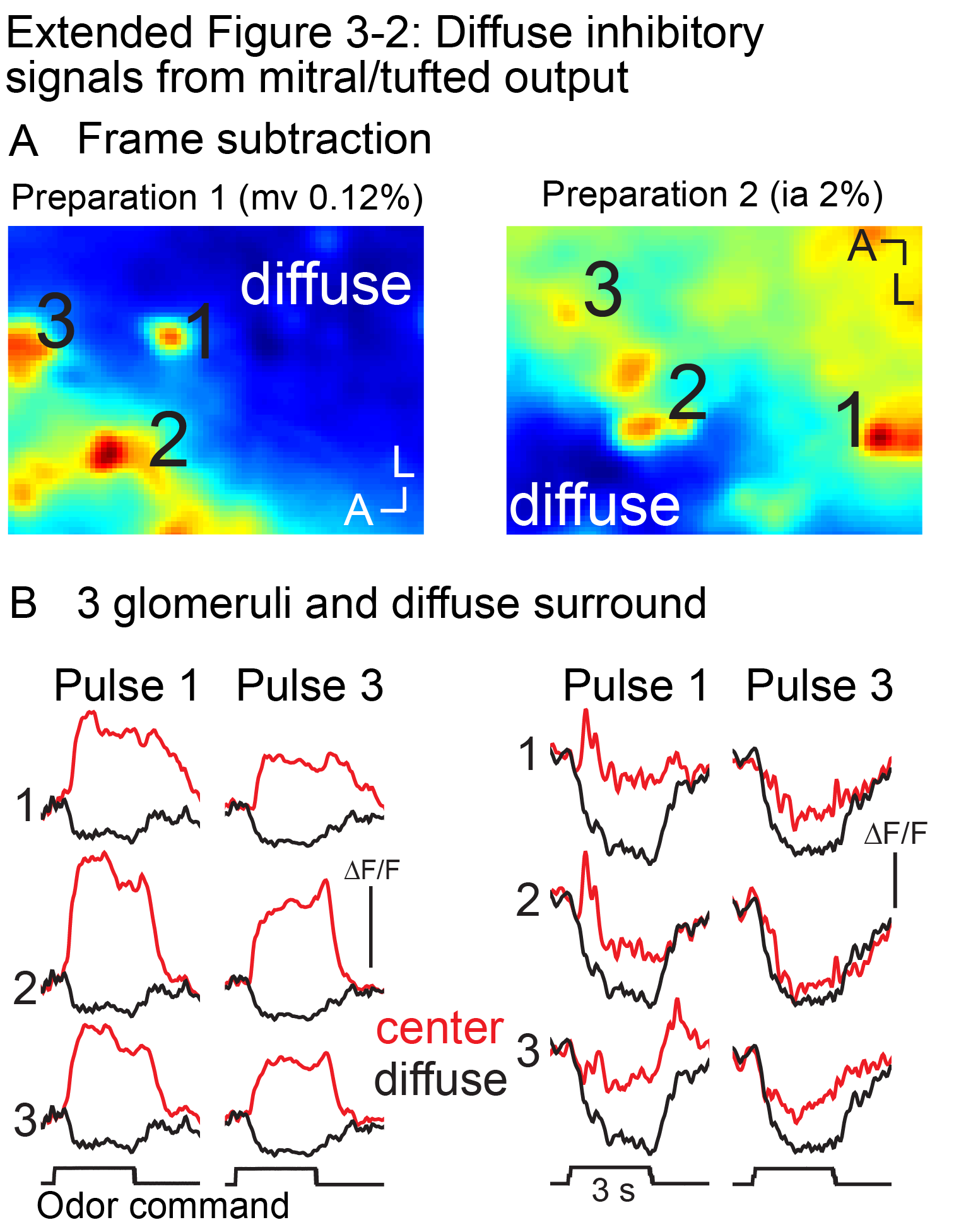

Supplement: Extended Data Figure 3-2 — The mitral/tufted output exhibited suppressed signals in response to odor stimulation in regions away from activated glomeruli. Two different preparations are shown from a Thy1-GCaMP6f 5.11 (left) transgenic mouse and a Tbx21-GCaMP6f (right) transgenic mouse. A, Activity maps in response to the initial odor presentation. The stimuli were methyl valerate at 0.12% of saturated vapor (left) and isoamyl acetate at 2% of saturated vapor (right). B, The center response of three glomeruli from each activity map (location indicated in A) along with the signal from the diffuse suppressed area. The glomerular center declined with repeated odor presentation, while the suppressed regions did not notably change. The scale bars in panel B indicate 10% (left) and 5% (right) ΔF/F, respectively. A, anterior; L, lateral. Download Figure 3-2, TIF file. [file enu-eN-NWR-0322-21-s09.tif]
